# Rapid genotyping by low-coverage resequencing to construct genetic linkage maps of fungi: a case study in *Lentinula edodes*

**DOI:** 10.1186/1756-0500-6-307

**Published:** 2013-08-02

**Authors:** Chun Hang Au, Man Kit Cheung, Man Chun Wong, Astley Kin Kan Chu, Patrick Tik Wan Law, Hoi Shan Kwan

**Affiliations:** 1 School of Life Sciences, The Chinese University of Hong Kong, Shatin, Hong Kong; 2 Core Facilities, Genome Sequencing Laboratory, The Chinese University of Hong Kong, Shatin, Hong Kong

**Keywords:** Assembly, Basidiomycota, Mapping, Mushroom, NGS, Pyrosequencing, QTL, Shiitake

## Abstract

**Background:**

Genetic linkage maps are important tools in breeding programmes and quantitative trait analyses. Traditional molecular markers used for genotyping are limited in throughput and efficiency. The advent of next-generation sequencing technologies has facilitated progeny genotyping and genetic linkage map construction in the major grains. However, the applicability of the approach remains untested in the fungal system.

**Findings:**

Shiitake mushroom, *Lentinula edodes*, is a basidiomycetous fungus that represents one of the most popular cultivated edible mushrooms. Here, we developed a rapid genotyping method based on low-coverage (~0.5 to 1.5-fold) whole-genome resequencing. We used the approach to genotype 20 single-spore isolates derived from *L*. *edodes* strain L54 and constructed the first high-density sequence-based genetic linkage map of *L*. *edodes*. The accuracy of the proposed genotyping method was verified experimentally with results from mating compatibility tests and PCR-single-strand conformation polymorphism on a few known genes. The linkage map spanned a total genetic distance of 637.1 cM and contained 13 linkage groups. Two hundred sequence-based markers were placed on the map, with an average marker spacing of 3.4 cM. The accuracy of the map was confirmed by comparing with previous maps the locations of known genes such as *matA* and *matB*.

**Conclusions:**

We used the shiitake mushroom as an example to provide a proof-of-principle that low-coverage resequencing could allow rapid genotyping of basidiospore-derived progenies, which could in turn facilitate the construction of high-density genetic linkage maps of basidiomycetous fungi for quantitative trait analyses and improvement of genome assembly.

## Findings

### Background

Genetic linkage maps are maps that show the relative positions and distances between markers or genes along the chromosomes as determined by their recombination frequency. Such maps are important in breeding programmes as they facilitate discovery of quantitative trait loci (QTL), association analysis as well as map-based localization and cloning of genes encoding agronomically essential phenotypes. However, the usefulness of a genetic linkage map depends on the choice and number of polymorphic markers used [1]. Traditional molecular markers used for genotyping purpose are limited in throughput and efficiency [2]. The advent of high-throughput next-generation sequencing (NGS) technologies has facilitated genotyping of progenies and construction of high-density genetic linkage maps in major grains such as rice [2,3], maize [4] and sorghum [5]. However, it remains unclear whether the approach is also applicable in the fungal system.

Shiitake mushroom, *Lentinula edodes*, is a basidiomycetous fungus that represents one of the most popular cultivated edible mushrooms. It also plays important roles in the pharmacological [6] and biotechnological fields [7]. *L*. *edodes* follows a typical basidiomycete life cycle, in which two monokaryotic mycelia with compatible mating types first fuse to form a dikaryon, the mycelia of which then aggregate to form a primordium under appropriate environmental conditions, which finally mature into a fruiting body. Currently available genetic linkage maps of *L*. *edodes* were constructed mainly using the fingerprinting type of markers such as randomly amplified polymorphic DNA (RAPD) [8-10] or amplified fragment length polymorphism (AFLP) markers [11,12] that are strain-specific, difficult to reproduce and lack DNA sequence information. Also, these maps are often of low-density. A high-density sequence-based genetic linkage map of *L*. *edodes* is currently unavailable.

Previously, we sequenced the draft genome of *L*. *edodes* monokaryon L54A by Roche 454 GS FLX-Titanium and ABI SOLiD sequencing at over 20-fold coverage [13]. The availability of the genome sequence provides the opportunity for rapid genotyping using the whole-genome resequencing approach. Here, we developed a rapid genotyping method by low-coverage resequencing on the 454 sequencing platform. We used the approach to genotype 20 single-spore isolates (SSIs) derived from *L*. *edodes* strain L54 and constructed, for the first time, a high-density sequence-based genetic linkage map of *L*. *edodes*. We used this as an example to provide a proof-of-principle that low-coverage resequencing could allow rapid genotyping of basidiospore-derived progenies for construction of high-density genetic linkage maps of basidiomycetous fungi.

### Results

The draft genome of *L*. *edodes* monokaryon L54A we sequenced previously contained 767 scaffolds after de novo genome assembly [13]. The total length of assembly was 40.2 Mb (92.4% in scaffolds). In this study, we resequenced the genomes of 20 SSIs from L54 in low coverage (~0.5 to 1.5-fold) (Additional file 1: Figure S1) using high-throughput 454 pyrosequencing. An average of 91,316 reads (range: 50,084–152,111) were obtained for each SSI, with a mean length of 378 bp (range: 367–392). About 77.2% of the reads per SSI were mapped on the reference genome (range: 74.9–81.2%). Chimeric and repetitive reads comprised 14.3% (range: 12.0–17.0%) and 5.3% (range: 4.5–5.9%), respectively, and were removed from subsequent analyses. About 2% of reads were unmapped (range: 1.0–3.0%). The mapped reads together covered 726 scaffolds (94.7%) of the reference genome. By considering a total of 186,079 single-nucleotide polymorphism (SNP) positions, ~75% of SSIs were successfully genotyping per scaffold (range: 5–100%), which were then used for the construction of a high-density sequence-based genetic linkage map.

**Figure 1:**
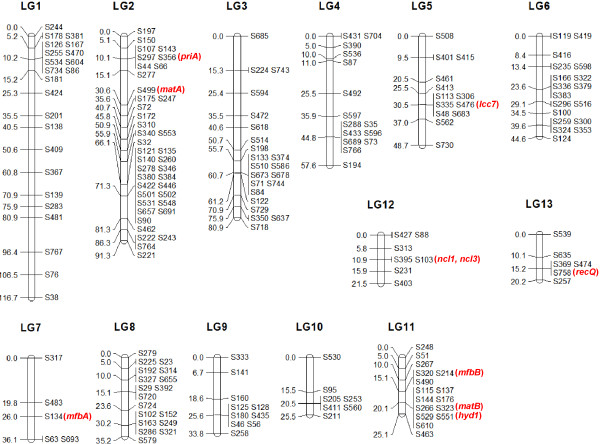
**Genetic linkage map of *L. edodes.*** The map was constructed using 20 SSIs. Linkage group numbers were indicated on the top, distances between markers (in cM) were shown on the left and the markers named according to the scaffolds were shown on the right. Genes discussed in the text were marked in red and placed next to the scaffold markers on which they resided.

The genetic linkage map spanned a genetic distance of 637.1 cM, and was composed of 13 linkage groups >20 cM in length (Figure 1, Table 1). The average size of the linkage groups was 49.0 cM. Two hundred sequence-based markers were placed on the linkage map. There were at least five and at most 40 sequence-based markers on the linkage groups, with an average of 15.4 markers per group. The distribution of the markers on the linkage groups was uneven. There were, for instance, 16 markers on the 25.1 cM long LG11, and only five markers were located on the 36.1 cM long LG7. The average spacing between markers varied from 1.7 cM to 9.0 cM, and the mean value was 3.4 cM. The total length of scaffolds on the linkage map was 18.8 Mb, covering 46.8% of the L54 genome. The average physical distance per linkage group was 1.4 Mb and the average physical to genetic distance ratio was 30.7 kbp/cM.

**Table 1:** Characteristics of the linkage groups

**Linkage group**	**Length (cM)**	**No. of markers**	Average marker spacing (cM)*	**Physical distance (kbp)**^**#**^	**Physical to genetic distance ratio (kbp/cM)**
LG1	116.7	23	5.3	2258.4	19.4
LG2	91.3	40	2.3	5185.9	56.8
LG3	80.9	22	3.9	302.9	3.7
LG4	57.6	15	4.1	1376.4	23.9
LG5	48.7	13	4.1	1574.6	32.3
LG6	44.6	18	2.6	1918.7	43.0
LG7	36.1	5	9.0	477.5	13.2
LG8	35.2	18	2.1	1948.7	55.4
LG9	33.8	10	3.8	956.1	28.3
LG10	25.5	7	4.3	533.4	20.9
LG11	25.1	16	1.7	801.8	32.0
LG12	21.5	7	3.6	667.5	31.0
LG13	20.2	6	4.0	800.7	39.7
Total	637.1	200	NA	18802.4	NA
Average	49.0	15.4	3.4^	1446.3	30.7

* Calculated by dividing the length of a particular linkage group by the number of markers on that linkage group minus one. ^ Calculated by dividing the size of the map by the total number of markers minus the number of linkage groups. ^#^ Calculated by adding up the size of all the scaffolds composing a particular linkage group.

The accuracy of the proposed genotyping method was verified experimentally (Additional file 2: Table S1). The genotypes of *priA* and *hyd1* determined by the current approach were in concordance with those determined by PCR-SSCP (single-strand conformation polymorphism) in all the 14 SSIs examined. Mating compatibility tests also confirmed all but one of the genotypes of the mating-type genes *matA* and *matB*. Therefore, the overall accuracy of the proposed genotyping approach was over 98%.

### Discussion

**Figure 2:**
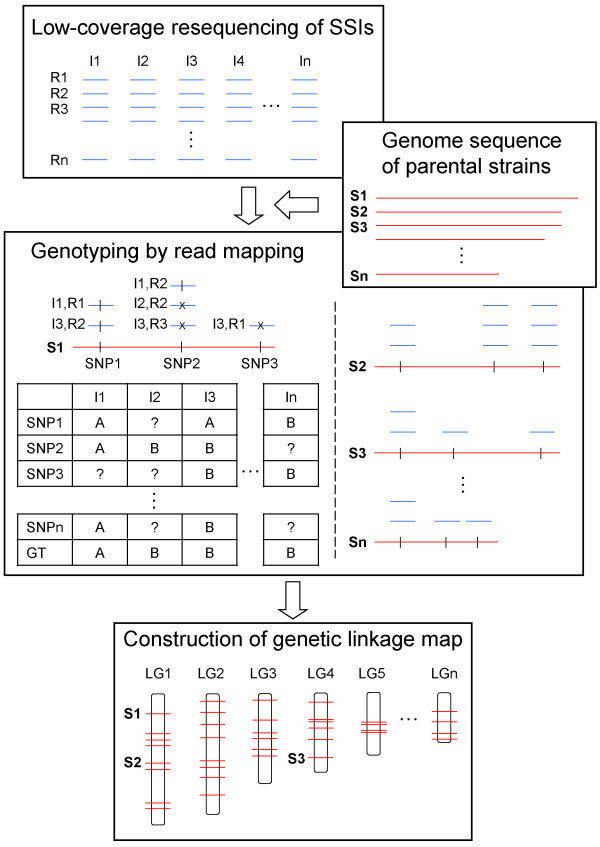
**Flowchart of the proposed genotyping approach.** Low-coverage (~0.5 to 1-fold) resequencing of a selected mapping population, here single-spore isolates (SSIs) I1 to In, is first performed on a next-generation sequencing (NGS) platform such as Roche 454, generating a library of short shotgun reads R1 to Rn for each isolate. Genotyping of the SSIs is then carried out by mapping the reads of each SSI to the draft parental reference genomes, represented in scaffolds S1 to Sn here. On each scaffold, identical mapped reads are assigned the same genotype of the parental strain being mapped whereas reads with high-quality single-nucleotide polymorphisms (SNPs) that are present in the other parent are assigned the opposite genotype. For each SSI, the final genotype (GT) of each scaffold, serving as a genetic marker, is called by a simple majority vote. All the scaffolds are genotyped in this manner. A genetic linkage map, on which linkage information of the markers on a set of linkage groups (LGs) is displayed, is then built based on the genotype segregation ratio of every marker examined.

Genotyping with various types of traditional molecular markers has been laborious and time-consuming. Sequencing simultaneously a large number of samples in multiplex on NGS platforms allows rapid and more cost-effective genotyping of whole mapping populations. Huang et al. [3] genotyped 150 recombinant inbred lines (RILs) of rice using low-coverage (~0.02-fold) resequencing on an Illumina Genome Analyzer platform. Rapid genotyping of 244 sorghum RILs was also achieved using ~0.07-fold resequencing [5]. Here, we developed a method to rapidly genotype basidiospore-derived progenies of *L*. *edodes* by low-coverage resequencing in ~0.5 to 1.5-fold (Figure 2). The approach could very likely be applied on other fungi. However, as the current approach requires genome data from the parental line, for fungal species lacking such information, the parent-independent genotyping approach developed by Xie et al. [2] should be employed instead.

To verify the accuracy of the proposed genotyping approach, we compared the genotypes of four known genes called using the current method with those determined experimentally using either mating compatibility tests or PCR-SSCP (Additional file 2: Table S1). Most of the results were concordant between the methods, except that the genotype of *matA* in SSI 9 was miscalled as “A” compared to “B” as determined by the mating compatibility tests. The *matA* locus was located on S499 which was >260 kbp in size (Additional file 3: Dataset S1). The current genotyping approach assigned a “A” genotype to the scaffold in SSI 9 based on a simple majority vote of genotype A:B = 292:131 (Data not shown). However, in-depth investigations revealed that the *matA* locus was located on a turning point before which the dominant genotype was “A” but at and after which the “B” genotype dominated. Indeed, a “B” genotype should be assigned to *matA* in SSI 9 if only reads mapped to the gene locus were counted (A:B = 1:13) (Data not shown). This probably represents a limitation of the current genotyping method, which is based on simple majority voting. The issue may be more prevalent in large scaffolds such as S499 here. As such, it is recommended to examine large scaffolds in more detail if genotypes of individual genes are to be investigated. However, as the overall estimated accuracy of the proposed approach was over 98%, we expect limited effects of the issue on the linkage relationships revealed between the scaffold markers.

The genetic linkage map constructed in this study composed 13 linkage groups (Figure 1). This is similar to that reported by Kwan and Xu [8], in which 14 linkage groups were found (Additional file 4: Table S2). However, only 11 linkage groups were revealed in some other studies [9-12]. A larger number of linkage groups observed is likely a result of a small mapping population used which affects the power to detect linkage. This is evidenced by the fact that several genes located on different linkage groups in this study were found on the same linkage groups in other studies. An example includes the nuclease genes *ncl1* and *ncl3*, which were both located on S103 of LG12 here (Figure 1, Additional file 3: Dataset S1). The two genes were on LGII, together with *priA* and *matA*, which were located on LG2 here, in a previous study [12]. This suggests that LG12 (21.53 cM long) may be linked with LG2 (91.34 cM long) to form LGII (161.3 cM long) reported previously. Another example consists of the *mfbA* and *recQ* genes, which were located on S134 of LG7 and S758 of LG13 in this study, respectively. These two genes were found on LG4 in another previous study [9], suggesting a linkage between LG7 (36.14 cM long) and LG13 (20.17 cM long) to form LG4 (92.5 cM long) reported previously. These further linkages will reduce the total number of linkage groups recovered here to 11. Nevertheless, neither of the before-mentioned maps seems to be saturated as the number of linkage groups does not agree with the haploid number of chromosomes: previous light microscopic observation [14] and electrophoretic karyotyping analysis [15] suggested that there are only eight chromosomes in *L*. *edodes*. Additional works have to be performed to confirm the actual number of chromosomes of *L*. *edodes* and to match the linkage groups with the respective chromosomes.

To examine the accuracy of the current linkage map, we compared with previous maps the locations of some known genes on the linkage groups. The *matA* locus was located on LGIII in Kwan & Xu [8], LGII in Terashima et al. [11,12] and LG1 in Miyazaki et al. [9,10], all of which were >90 cM in length. In this study, the *matA* locus was located on S499 (30.6 cM) on LG2 (Figure 1, Additional file 3: Dataset S1), the linkage group with the longest physical length (~5.2 Mb) and second longest genetic distance (91.3 cM) (Table 1). This is in agreement with the previous findings. The *matA* and *priA* loci are always found on the same linkage group [8-10,12]. The same also held in our map, in which *priA* was located on S356 (10.1 cM) on LG2. The *matB* locus was on LGII in Kwan and Xu [8], LGV in Terashima et al. [11,12] and LG11 in Miyazaki et al. [9,10], and was on S323 (20.1 cM) on LG11 in this study. The co-existence of *matB* and *hyd1* on the same linkage group reported previously [9,10,12] was also recovered here. The same is also true for the co-existence of *matB*, *hyd1* and *mfbB* on the same linkage group reported in Miyazaki et al. [9,10]. Overall, the genetic linkage map constructed here seems to be in good agreement with other currently available maps.

A high-density genetic linkage map could enhance the precision of QTL mapping. Miyazaki et al. [10] constructed a linkage map of *L*. *edodes* that comprised 301 markers and spanned ~900 cM (Additional file 4: Table S2), representing the densest map of *L*. *edodes* at that time. However, only 35 of the markers in that study were sequence-based. The actual usefulness of the map in, for instance, QTL analyses is thus very limited. Here, 200 sequence-based markers were placed on a genetic linkage map of *L*. *edodes*. The total genetic distance of the map was ~640 cM, and the average marker spacing was 3.4 cM. It represents the densest map of *L*. *edodes* that is based on sequence-based markers at present. However, our map is still inadequate for accurate trait mapping as it involves only a small mapping population of 20 SSIs. For accurate trait mapping, a larger mapping population should be recruited.

Although accurate trait mapping is infeasible here, the presence of some gene clusters might provide insights on some phenotypic traits. For instance, the experimentally expressed laccase gene *lcc7* was located on S476 (30.5 cM) of LG5 here, and in linkage with genes encoding P450 monooxygenase and oxidoreductase, which were on S48 on the same location of the same linkage group (Figure 1, Additional file 3: Dataset S1). These genes, together with some others, formed a cluster that controls the biosynthesis of the pigment aurofusarin in the plant pathogenic filamentous fungus *Fusarium graminearum*[16]. Indeed, an up-regulated expression of these genes was observed in the brown film-forming mycelium of *L*. *edodes*[17]. These suggest the potential importance of the gene cluster in pigment production of *L*. *edodes*, although more detailed trait mapping experiments are needed for confirmation, but this is out of the scope of the present study.

Apart from facilitating quantitative trait analyses, a high-density genetic linkage map could also aid genome assembly. Repetitive sequences represent one of the biggest difficulties in genome assembly. This is especially in the case of fungi – fungal genomes typically contain 3-10% of repeat contents [18]. By linking the physical map (genome sequence) to the genetic linkage map, genetics-based superscaffolds could be formed, allowing the anchoring and ordering of scaffolds involved. Here, 200 scaffolds representing 46.8% of the L54 genome were mapped on the 13 linkage groups in the current genetic linkage map of *L*. *edodes* (Table 1). As the density of markers on a linkage map is limited by the number of recombination events occurring in meiosis of the mapping population, it is expected that an even denser map on which more scaffolds reside could be generated with a larger mapping population using the current approach.

### Conclusions

Based on low-coverage resequencing of 20 SSIs derived from *L*. *edodes* strain L54, a high-density sequence-based genetic linkage map of *L*. *edodes* was obtained for the first time. We provided a proof-of-principle that low-coverage resequencing could allow rapid genotyping of basidiospore-derived progenies for construction of high-density genetic linkage maps of basidiomycetous fungi. Follow-up studies with a larger mapping population could allow generation of a denser genetic linkage map of *L*. *edodes* that could in turn facilitate QTL identification, breeding programme monitoring as well as genome assembly.

### Materials and methods

#### SSI cultivation and DNA extraction

Twenty basidiospores were randomly isolated from fresh-fruiting bodies of the *L*. *edodes* dikaryotic L54 strain. Monokaryotic mycelia were cultured in liquid potato dextrose broth (PDB) and grown for 1–2 months at 25°C in the dark. The mycelia were collected by filtration, frozen and homogenized in liquid nitrogen. Genomic DNA was extracted from the mycelial fragments using the DNeasy Plant Mini Kit (Qiagen).

#### Shotgun sequencing and genotyping

Shotgun sequencing of the 20 SSIs was performed in multiplex with multiplex identifiers (MIDs) on a GS FLX-Titanium sequencer (454 Life Sciences Corporation) in 1.5 plates run. Genotyping was performed by mapping the sequencing reads of each SSI to the parental L54A reference genome using the GS Reference Mapper version 2.6 (454 Life Sciences Corporation) with default parameters. Identical mapped reads were assigned to genotype “A” whereas reads with high-quality SNPs were regarded as genotype “B”. Here, each scaffold sequence (genotype A or B) served as a genetic marker for linkage map construction. The genotype profile of markers for each SSI was generated using simple majority voting. Briefly, on a certain scaffold, if a particular SSI contained more mapped reads with genotype “A” than “B”, then the marker was genotyped as “A”, and vice versa. A “X” was assigned in cases where there were an equal number of “A” and “B”, and a “-“was assigned when no reads were mapped on the scaffold. The genotyping process was automated using in-house developed Perl scripts. A flowchart of the procedure was given in Figure 2.

#### Genetic linkage map construction

The genetic linkage map was built using MSTMap [19]. Genotype segregation ratio of every marker was examined using the *χ*^2^ test. Markers with a segregation ratio of 1:1 (P < 0.05) were clustered into linkage groups when the two-point LOD scored ≥ 3. Map distances between markers were calculated based on the Kosambi’s mapping function. The maximum distance between markers in a single linkage group allowed was 25 centiMorgan (cM). Orientation of markers within individual linkage groups was confirmed by feeding smaller marker subsets to MAPMAKER/EXP V3.0 [20]. The genetic linkage map was drawn using MapChart V2.2 [21].

#### Mating compatibility tests and PCR-SSCP

To verify the accuracy of the proposed genotyping approach, the genotypes of *matA*, *matB*, *priA*, and *hyd1* were determined experimentally in 14 randomly selected SSIs (Additional file 2: Table S1). Mating compatibility tests were carried out to determine the genotypes of the mating-type genes *matA* and *matB* as described previously [8]. Briefly, mating reactions were performed between each of the SSIs and the parents, as well as between all of the SSIs. In each cross, two plugs of agar and mycelia from two monokaryons were placed ~1.5 cm apart on a petri dish that contained a potato dextrose agar (PDA) medium, and cultivated for 1–2 weeks at 25°C in the dark. Mycelial interactions were observed. The formation of clamp connections in a cross suggests opposite genotypes of both the mating-type genes shared between the crossed individuals.

PCR-SSCP was used to determine the genotypes of *priA* and *hyd1*. In brief, amplifications of the genes were carried out with PCR primers designed based on reference sequences on GenBank (Additional file 5: Protocol S1), and SSCP between the PCR products of the SSIs and the parental strains was tested using the GeneGel Exel 12.5/24 Kit (GE Healthcare) following the manufacturer’s instructions. Electrophoresis was carried out on a GenePhor Electrophoresis Unit (GE Healthcare), and silver staining of the polyacrylamide gels was performed using the Hoefer Automated Gel Stainer with the PlusOne DNA Silver Staining Kit (GE Healthcare). Individuals sharing the same SSCP pattern were regarded as having the same genotype.

## Availability of supporting data

The data set supporting the results of this article is available in the NCBI Sequence Read Archive under the accession number SRP026195 (http://www.ncbi.nlm.nih.gov/sra/?term=SRP026195).

## Abbreviations

AFLP: Amplified fragment length polymorphism; cM: centiMorgan; MID: Multiplex identifier; NGS: Next-generation sequencing; PDA: Potato dextrose agar; PDB: Potato dextrose broth; QTL: Quantitative trait loci; RAPD: Randomly amplified polymorphic DNA; RIL: Recombinant inbred line; SNP: Single-nucleotide polymorphism; SSCP: Single-strand conformation polymorphism; SSI: Single-spore isolate.

## Competing interests

The authors declare that they have no competing interests.

## Authors’ contributions

CHA performed genotyping and genetic linkage map construction and helped draft the manuscript. MKC participated in genetic linkage map construction and wrote the manuscript. MCW was responsible for SSI cultivation and DNA extraction. AKKC carried out the mating compatibility tests and PCR-SSCP analysis. PTWL performed genome sequencing. HSK conceived and supervised the study. All authors read and approved the final manuscript.

## Supplementary Material

Additional file 1: Figure S1 Whole-genome resequencing throughput of the SSIs, Description. The genome size of the reference *L*. *edodes* L54A strain is 40.2 Mb. Click here for file

Additional file 2: Table S1 Verification of the genotyping results. Description: Genotypes before the slashes were determined experimentally whereas those after the slashes were determined by the proposed genotyping approach. The genotype in parentheses could be corrected after manual curation. Click here for file

Additional file 3: Dataset S1 Example of genes on the linkage map markers. Description: Examples of genes located on each of the linkage map markers. Click here for file

Additional file 4: Table S2 Comparison of major *L*. *edodes* genetic linkage maps. Description: Comparison of characteristics of major *L*. *edodes* genetic linkage maps. Click here for file

Additional file 5: Protocol S1 PCR primers and protocol for PCR-SSCP. Description: Detailed protocol for PCR-SSCP. Click here for file
