# Supplementary figures and images for: Rapid genotyping by low-coverage resequencing to construct genetic linkage maps of fungi: a case study in Lentinula edodes
Source: BMC Res Notes. 2013 Aug 2;6:307. doi: 10.1186/1756-0500-6-307 (PMC3750829; doi:10.1186/1756-0500-6-307)

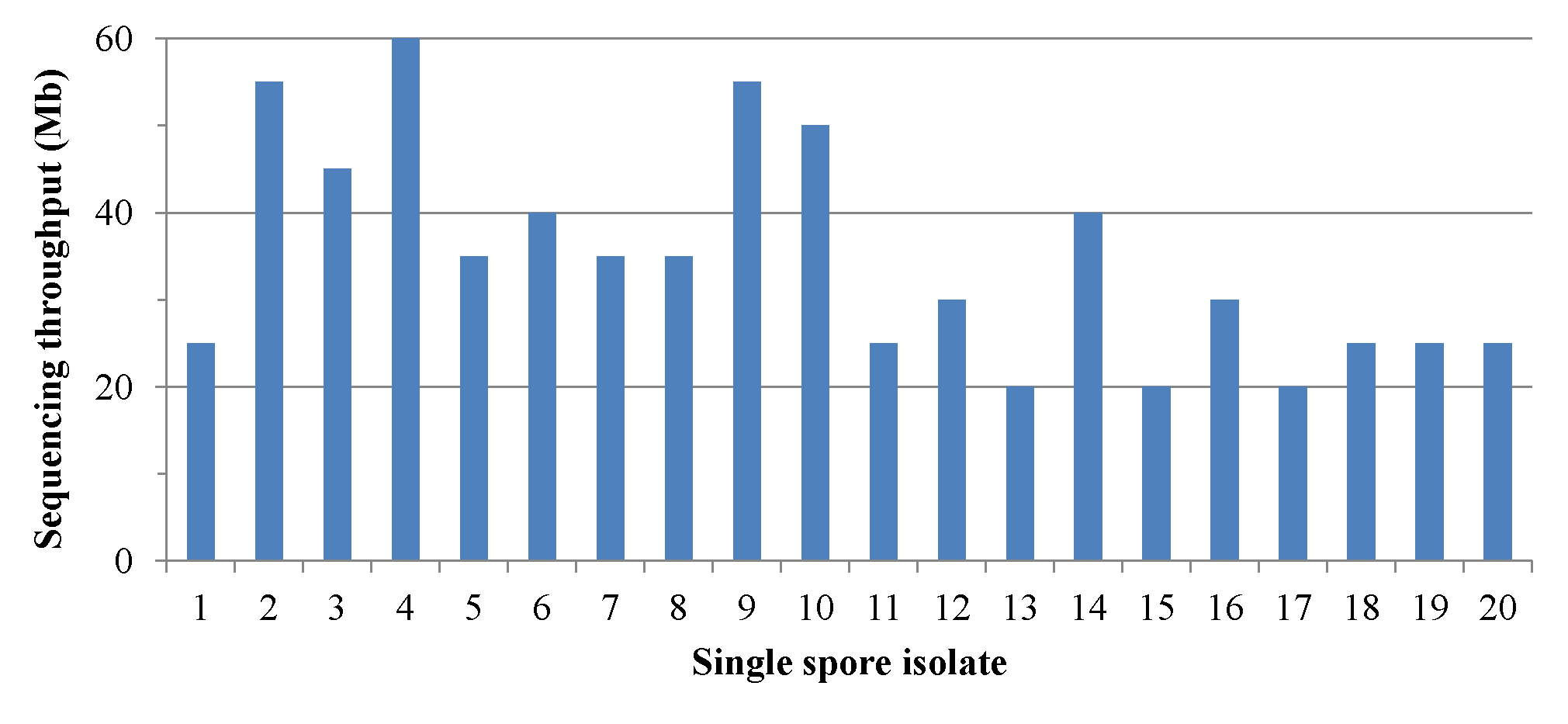

Supplement: Additional file 1: Figure S1 — Whole-genome resequencing throughput of the SSIs, Description. The genome size of the reference L. edodes L54A strain is 40.2 Mb. [file 1756-0500-6-307-S1.png]
