# Supplementary material for: Rapid genotyping by low-coverage resequencing to construct genetic linkage maps of fungi: a case study in Lentinula edodes
Source: BMC Res Notes. 2013 Aug 2;6:307. doi: 10.1186/1756-0500-6-307 (PMC3750829; doi:10.1186/1756-0500-6-307)
Supplement: Additional file 2: Table S1 — Verification of the genotyping results. Description: Genotypes before the slashes were determined experimentally whereas those after the slashes were determined by the proposed genotyping approach. The genotype in parentheses could be corrected after manual curation. [file 1756-0500-6-307-S2.doc]

**Table S1. Verification of the genotyping results.** Genotypes before the slashes were determined experimentally whereas those after the slashes were determined by the proposed genotyping approach. The genotype in parentheses could be corrected after manual curation.

| **SSI** | ***matA**** | ***priA^*** | ***hyd1^*** | ***matB**** |
| --- | --- | --- | --- | --- |
| 1 | B/B | B/B | A/A | A/A |
| 2 | B/B | B/B | B/B | B/B |
| 3 | A/A | A/A | A/A | A/A |
| 4 | B/B | B/B | A/A | A/A |
| 5 | A/A | A/A | B/B | B/B |
| 6 | A/A | A/A | B/B | B/B |
| 8 | A/A | A/A | B/B | B/B |
| 9 | B/(A) | A/A | A/A | A/A |
| 10 | A/A | A/A | A/A | A/A |
| 11 | B/B | B/B | B/B | B/B |
| 12 | A/A | A/A | A/A | A/A |
| 13 | A/A | A/A | B/B | B/B |
| 14 | B/B | B/B | B/B | B/B |
| 15 | B/B | A/A | A/A | A/A |

* Based on mating compatibility tests.

^ Based on PCR-SSCP.
