# Supplementary material for: Rapid genotyping by low-coverage resequencing to construct genetic linkage maps of fungi: a case study in Lentinula edodes
Source: BMC Res Notes. 2013 Aug 2;6:307. doi: 10.1186/1756-0500-6-307 (PMC3750829; doi:10.1186/1756-0500-6-307)
Supplement: Additional file 4: Table S2 — Comparison of major L. edodes genetic linkage maps. Description: Comparison of characteristics of major L. edodes genetic linkage maps. [file 1756-0500-6-307-S4.pdf]

Table S2. Comparison of major *L. edodes* genetic linkage maps.

Reference	Mapping population size	Map size (cM)	No. of linkage groups	No. of markers	Average marker spacing (cM)*	No. of sequence-based markers
[8]	32	622.4	14	69	11.3	2
[11]	95	1956.7	11	206	10.0	0
[12]	95	1398.4	11	166	9.0	10
[9]	92	908.8	11	289	3.3	23
[10]	92	908.8	11	301	3.1	35
**This study**	**20**	**637.1**	**13**	**200**	**3.4**	**200**

* Calculated by dividing the size of map by the number of markers minus the number of linkage groups.
