# Supplementary material for: Rapid genotyping by low-coverage resequencing to construct genetic linkage maps of fungi: a case study in Lentinula edodes
Source: BMC Res Notes. 2013 Aug 2;6:307. doi: 10.1186/1756-0500-6-307 (PMC3750829; doi:10.1186/1756-0500-6-307)
Supplement: Additional file 5: Protocol S1 — PCR primers and protocol for PCR-SSCP. Description: Detailed protocol for PCR-SSCP. [file 1756-0500-6-307-S5.doc]

**Protocol S1. PCR primers and protocol for PCR-SSCP.**

| **Gene marker** | **Primer** | **Sequence (5 'to 3')** | **GenBank reference** |
| --- | --- | --- | --- |
| *priA* | Upper Primer | CGTGCTTGCTCTTGCTAT | X60956 |
|  | Lower Primer | GACCCTTTCCGTTCAGAT |  |
| *hyd1* | Upper Primer | TGCCCGAGAGAACGATAC | CAA74987 |
|  | Lower Primer | CACCGCCTCTTCTCCTAC |  |

Each 20 µl PCR mix contained 2 µl of 10X DNA polymerase buffer (Promega), 2 µl of 25 mM MgCl_2_ solution, 0.4 µl of 10 mM dNTP mix, 2 µl of 10 µM upper primer, 2 µl of 10 µM lower primer, 1 µl of DNA template (~100 ng/µl), and 0.4 U of *Taq* polymerase (Promega). The PCR thermal regime consisted of an initial denaturation of 3 mins at 94^o^C, followed by 35 cycles of denaturation of 1 min at 94^o^C, annealing of 1 min at 55^o^C (for *priA*) or 51^o^C (for *hyd1*), and extension of 1 min at 72^o^C, and a final extension of 10 mins at 72^o^C. The PCR products were checked with electrophoresis on 1% TBE agarose gel in 1X TBE buffer with ethidium bromide staining.
